# Testing for Recent HIV Infections: A Conceptual Review

**DOI:** 10.3390/pathogens15101097

**Published:** 2026-10-21

**Authors:** Barbara Suligoi, Claudio Galli

**Affiliations:** 1 Comitato Tecnico Sanitario per la Lotta all’AIDS, Italian Ministry of Health, 00100 Roma, Italy; bsuligoi@gmail.com; 2 Independent Researcher, 00139 Roma, Italy

**Keywords:** HIV incidence, recency assays, LAg, STARHS, genomic incidence, surveillance, MDRI, FRR

## Abstract

Accurate HIV incidence estimation is essential for monitoring epidemic dynamics and guiding prevention strategies. Recency assays—laboratory-based or point-of-care tests that distinguish recent from long-standing HIV infection—have made cross-sectional incidence estimation increasingly feasible. This review synthesizes the evolution, validation, applications, limitations, and future directions of HIV recency assays, from early detuned assays to modern genomic approaches. We conducted a narrative synthesis of foundational methodological papers, validation studies, global guidance documents, and programmatic evaluations, including UNAIDS/WHO technical guidance and CEPHIA assay performance assessments. Recency assays have evolved through four major generations: detuned EIAs (STARHS), avidity-based assays, rapid point-of-care recency tests, and genomic incidence assays. Their performance is governed by two core metrics—mean duration of recent infection (MDRI) and false-recent rate (FRR)—which vary by subtype, ART exposure, host factors, and assay platform. Recency assays are now used in national surveys, routine HIV testing services, key-population surveillance, and outbreak detection. However, limitations include biological variability, ART-related misclassification, ethical concerns, and operational challenges. In conclusion, recency assays are powerful but imperfect tools. Their future lies in improved assay design, integration with prevention programs and trials, real-time surveillance systems, and convergence with genomic and program data. Ethical safeguards and community engagement remain essential as HIV recency testing becomes more embedded in public health practice.

## 1. Overview of Recent HIV Infections

HIV surveillance has traditionally relied on the reporting of newly diagnosed HIV infections as a key indicator of the magnitude and evolution of the epidemic. However, an important distinction should be made between a *new HIV diagnosis* and a *recent HIV infection*. A new diagnosis refers to the identification of HIV infection in an individual who was previously unaware of being infected, without necessarily providing information on when the infection was acquired. In contrast, recent HIV infection is defined according to the time elapsed since HIV acquisition and therefore aims to identify infections that occurred within a predefined period, commonly within the previous 6 or 12 months [1]. This distinction is epidemiologically important because the number of people newly diagnosed with HIV in a given year reflects not only infections acquired during that year, or shortly before it, but also infections acquired months or years earlier. WHO has consequently defined recent HIV infection as a state that begins at the moment HIV infection is initiated and whose duration can be defined either chronologically, for example, as six months after infection, or biologically on the basis of biomarkers that change over the course of infection [1].

**Figure 1:**
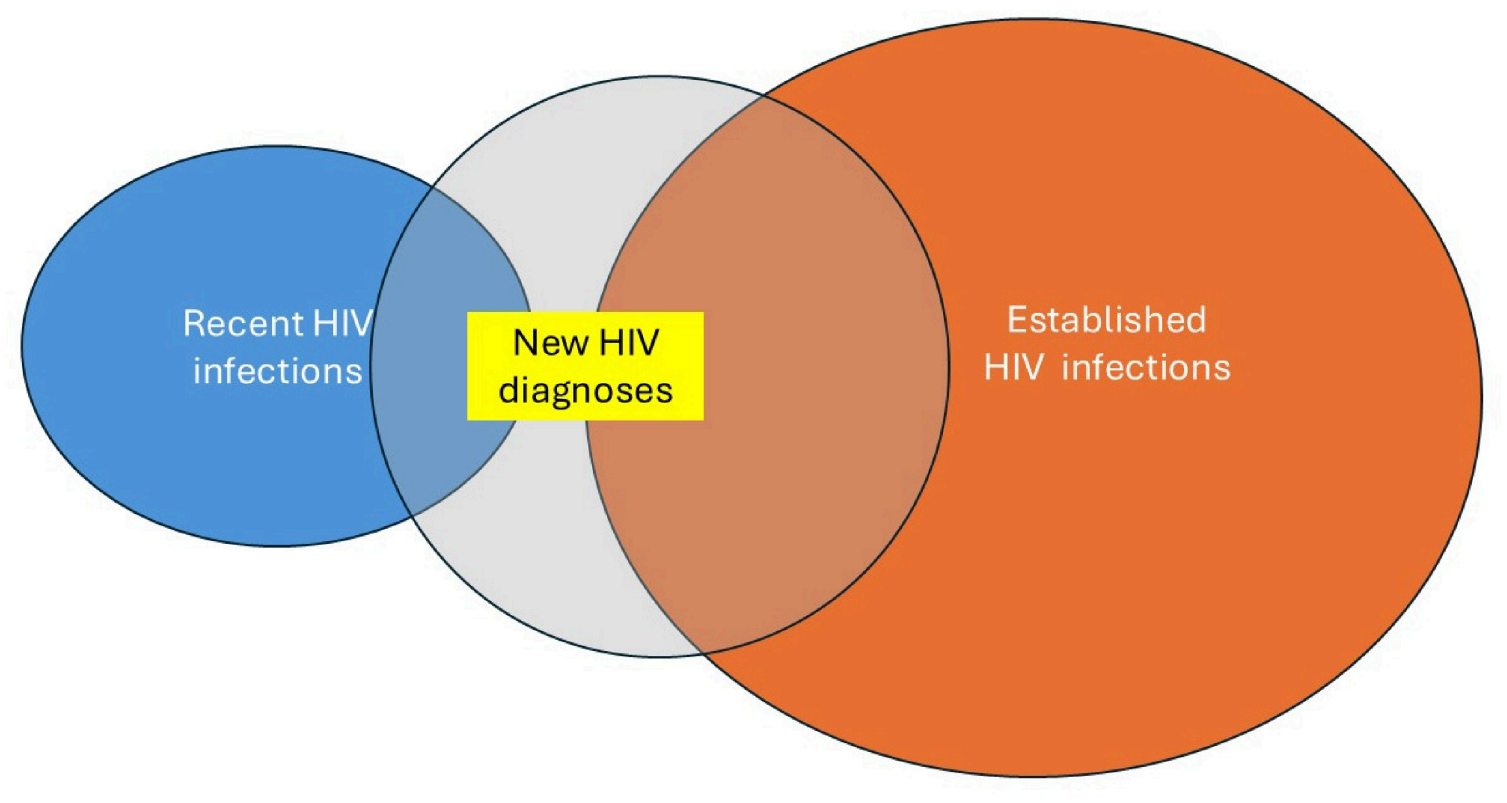
Venn diagram illustrating the distinction and overlap between recent HIV infections (defined by recency assays), new diagnoses, and established chronic infections (illustrative graph).

Routine HIV surveillance systems predominantly rely on case reporting and therefore describe the number and characteristics of people newly diagnosed with HIV rather than the number of people who have acquired HIV during a given period. Such data are indispensable for monitoring the burden of HIV, guiding healthcare provision and evaluating testing strategies, but they do not provide a direct measure of HIV incidence [1,2]. The distinction and overlap between recent HIV infections, new diagnoses, and established chronic infections must be clear and is particularly relevant in settings where late diagnosis remains common (Figure 1).

In Europe, for example, late diagnosis continues to represent a substantial proportion of all new HIV diagnoses. According to the most recent ECDC/WHO surveillance data, 48% of new HIV diagnoses reported in the European Union/European Economic Area (EU/EEA) in 2024 were made at a late stage of infection, defined by a CD4 cell count below 350 cells/mm^3^, while the corresponding proportion for the wider WHO European Region was 54% [3]. The high proportion of late diagnoses illustrates the temporal gap that may exist between HIV acquisition and diagnosis. A person diagnosed today may have acquired HIV relatively recently but may also have been infected several years earlier and remained undiagnosed during that period. Consequently, trends in annual HIV diagnoses cannot be interpreted straightforwardly as trends in the incidence of new infections. The epidemic described by routine diagnosis-based surveillance represents a mixture of infections acquired at different points in time. This temporal distortion is particularly relevant when evaluating the impact of prevention interventions, changes in transmission patterns, or emerging prevention strategies, because an increase or decrease in diagnoses may reflect changes in testing practices and case detection, as well as changes in HIV transmission itself [2]. Indeed, ECDC surveillance data have repeatedly shown that changes in testing policies and testing intensity can influence the number of diagnoses reported in a given period [3].

A more accurate assessment of the current dynamics of the HIV epidemic would therefore require information on the timing of infection in addition to the timing of diagnosis. However, determining the precise date of HIV acquisition in an individual remains challenging.

In this review, we summarize the approaches that have been developed to identify recent HIV infection, with particular attention to studies conducted over the past two decades. This is a brief, narrative review that, by definition, uses a flexible, qualitative approach to interpret, summarize, and synthesize the literature on a broad topic without a rigid protocol. Unlike a systematic review, a narrative review does not necessarily require an exhaustive database search or predefined inclusion and exclusion criteria or assessment of the risks of bias. We describe the main laboratory assays, biomarkers, and testing algorithms proposed to distinguish recently acquired from long-standing HIV infection and critically discuss their respective advantages and limitations. The literature included in this narrative review was selected by identifying the earliest published evidence on each recency assay or strategy, the most relevant review articles on the topic, and relevant documents published by ECDC, WHO, and UNAIDS. By synthesizing this evidence, the review aims to clarify the extent to which approaches to identifying recent infections can complement conventional HIV diagnosis-based surveillance and contribute to a timelier and more informative characterization of the HIV epidemic.

## 2. General Principles on HIV Recency Testing

There is currently no diagnostic test that can reliably establish the exact time elapsed since HIV infection. This limitation arises from the biological evolution of HIV infection and the dynamics of the host immune response. Following HIV acquisition, viral markers become detectable before or around the time of seroconversion, followed by progressive changes in the magnitude, specificity, and avidity of the antibody response. Although these changes can be exploited to distinguish recent from long-standing infections at a population level, they do not follow a sufficiently uniform trajectory among individuals to provide an exact infection date. Accordingly, no single laboratory test can currently be regarded as a gold-standard method for accurately determining the time since HIV acquisition in an individual newly diagnosed with HIV.

**Figure 2:**
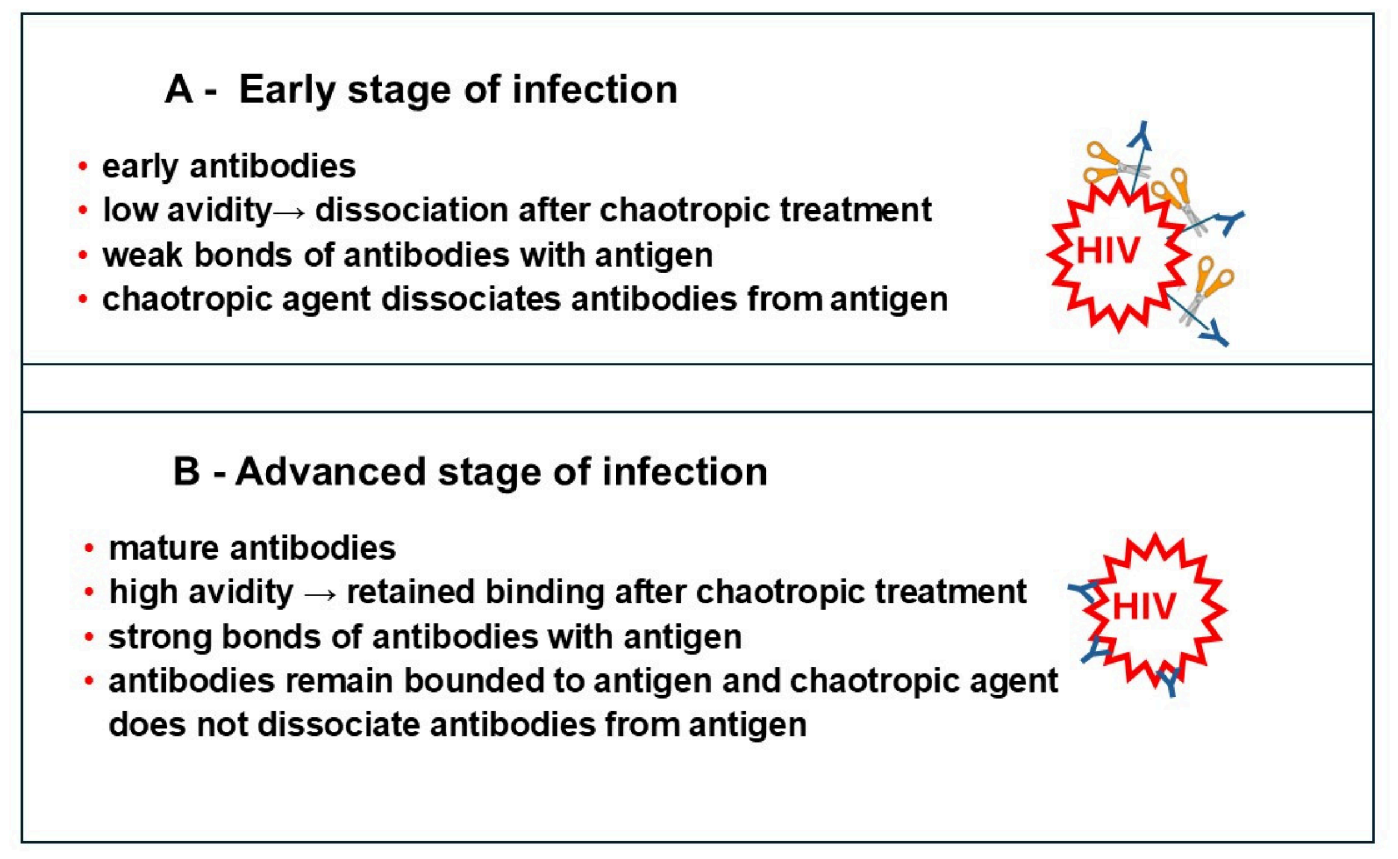
Principle of antibody avidity testing for distinguishing early and advanced HIV infection. 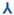 Antibody; 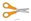 chaotropic agent that dissociates weak bonds between antigen and antibody.

Despite this limitation, considerable efforts have been made over the past two decades to identify laboratory and epidemiological markers capable of classifying HIV infections according to recency. The underlying principle is that certain characteristics of the immune response change progressively after infection and can therefore be used to identify individuals who are more likely to have acquired HIV within a predefined period. Several serological approaches have been developed, including assays based on antibody maturation, antibody avidity, and changes in antibody responses to specific HIV antigens. Assays that use low antibody avidity during the early stages of infection as a marker of recent infection are the most widely investigated (Figure 2).

Among these, the limiting-antigen (LAg) avidity assay is one of the most frequently applied approaches for identifying recent HIV infection and estimating HIV incidence at the population level [4]. Other approaches have included modifications of conventional diagnostic assays based on reduced sensitivity [5], antibody levels [6], or antibody avidity [7,8,9,10], as well as the evaluation of combinations of different serological markers [11,12].

Importantly, these approaches do not identify “recent infection” as an absolute biological state with a universally applicable duration. Instead, each assay identifies a period during which recently infected individuals are expected to be classified as recent; this is expressed as the mean duration of recent infection (MDRI). Assays can also misclassify some people with long-standing infection as recently infected; this is quantified by the false-recent rate (FRR). Considerable effort has therefore focused on improving assay performance and reducing misclassification, as well as developing testing algorithms that combine different laboratories and clinical parameters to improve the classification of recent infection [13,14,15].

The development of these methods has generated a substantial body of evidence over the last two decades. The Consortium for the Evaluation and Performance of HIV Incidence Assays (CEPHIA), for example, independently evaluated multiple candidate incidence assays and demonstrated considerable variability in assay performance, including the influence of viral subtype, viral suppression, and antiretroviral treatment [14,15,16]. These studies also highlighted a fundamental challenge in the development of HIV recency assays: achieving a sufficiently long MDRI while maintaining a very low FRR. Misclassification of long-standing infections may be particularly problematic among individuals who are virally suppressed, including those receiving effective antiretroviral therapy, and can substantially bias incidence estimates if not adequately addressed. HIV elite controllers as well can be misclassified by recency assays because their atypical immune control and very low untreated viral replication can produce biomarker profiles resembling those of early/recent HIV infection [17].

The available evidence therefore suggests that no single assay or biomarker can provide a definitive diagnosis of recent HIV infection in an individual. Rather, available approaches provide a probabilistic classification that is most useful when applied at the population level and interpreted within a well-defined epidemiological framework [1]. Their performance depends on the biological characteristics of the HIV infection, the assay used, the cutoff used, the definition of the recent period, the HIV subtype, treatment status, and the characteristics of the population being tested. These limitations have driven the development of RITAs and have also emphasized the importance of appropriate assay validation and quality assurance before implementation in surveillance programs [1,13].

Taken together, these considerations highlight a fundamental limitation of conventional HIV surveillance: counting new diagnoses provides essential information on the number of infections being detected by health systems but does not necessarily provide a contemporaneous picture of HIV transmission. Recent-infection testing has emerged as a complementary approach that adds a temporal dimension to surveillance, although its interpretation requires careful consideration of the biological, analytical, and epidemiological limitations of each method [1,18].

## 3. Biological Basis of Recent HIV Infection Testing

### 3.1. HIV Infection Staging

The identification of recent HIV infection is primarily based on the stages of early HIV infection. Following HIV acquisition, the infection progresses through several well-defined stages:

- Acute HIV infection: Characterized by extremely high viral loads and the absence of detectable antibodies. Symptoms may resemble influenza or mononucleosis [19,20].
- Recent (or early) HIV infection: Usually defined as the first several months after seroconversion. During this period, antibody avidity and concentration continue to mature [19,21,22].
- Established (or long-standing) infection: Antibody responses stabilize, and viral load often reaches a set point [20,22].

### 3.2. Virological Assays for HIV Recency

Recent-infection assays exploit the biological differences between recent and established infection, mostly based on the maturation of the antibody response. Several biomarkers have been explored for this purpose. Their rationale and limitations are described in Table 1.

## 4. Assay Performance Metrics of Recent HIV Infection Assays: MDRI and FRR

Two parameters are essential for interpreting recent infection assays, specifically:

- Mean duration of recent infection (MDRI): This is the average time an individual remains classified as “recent” by a given assay or algorithm. This parameter can be based either on the assay’s ability to discriminate early from long-term infections or the definition of “recent” infection selected by researchers (e.g., 3, 6, or 12 months). For many assays, MDRI ranges between 4 and 6 months.
- False-recent rate (FRR): This is the proportion of established infections misclassified as recent. An FRR of ≤2% has been considered a desirable target for HIV incidence assays, although the FRR should be empirically estimated for the specific assay, algorithm, and target population and may need to be incorporated into incidence calculations [18].

**Figure 3:**
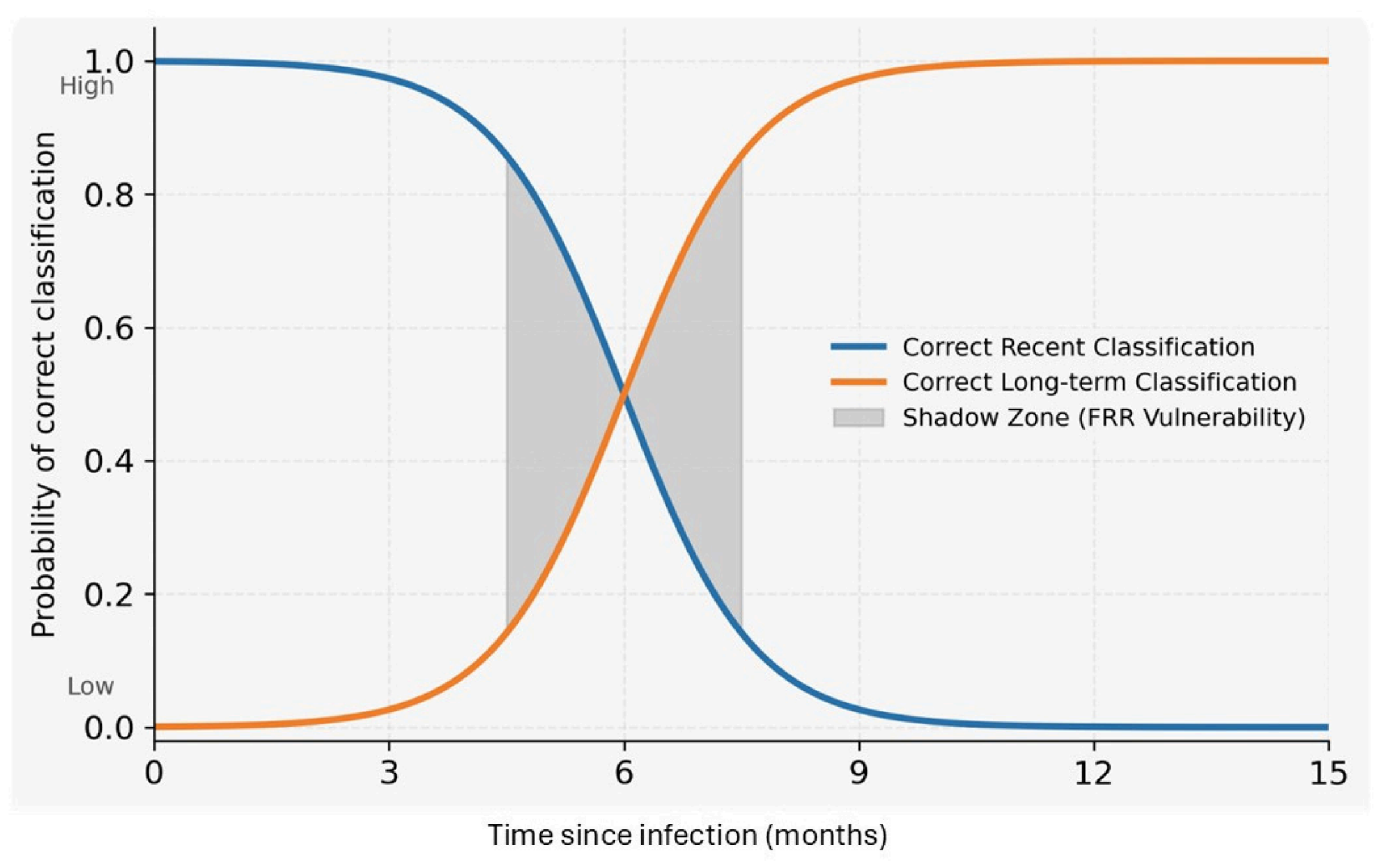
Mathematical performance profile of the HIV recency assay (illustrative graph). The blue line illustrates the probability of correct classification for recent infections, which declines as time since infection increases. Conversely, the orange line indicates the probability of correct classification for long-term infections. The shaded area highlights the transition window (“shadow zone”), where the assay is vulnerable to misclassification and false-recent rate (FRR) effects.

The probability of correct classification for recent infections using recency assays declines as time since infection increases. Conversely, the probability of correct classification of long-term infections increases with increasing time from infection (Figure 3). There is a transition window, or “shadow zone”, where the assay is vulnerable to misclassification and FRR effects.

Recency-assay cutoffs may require regional or HIV-clade-specific optimization because differences in subtype and population characteristics can affect the MDRI and FRR. Raising the recency threshold generally increases the MDRI but may also increase the FRR. Cutoffs should therefore be optimized using local or subtype-specific performance data to balance a longer recency window against the risk of falsely classifying long-standing infections as recent [18].

## 5. Major Assays for Recent HIV Infection

**Figure 4:**
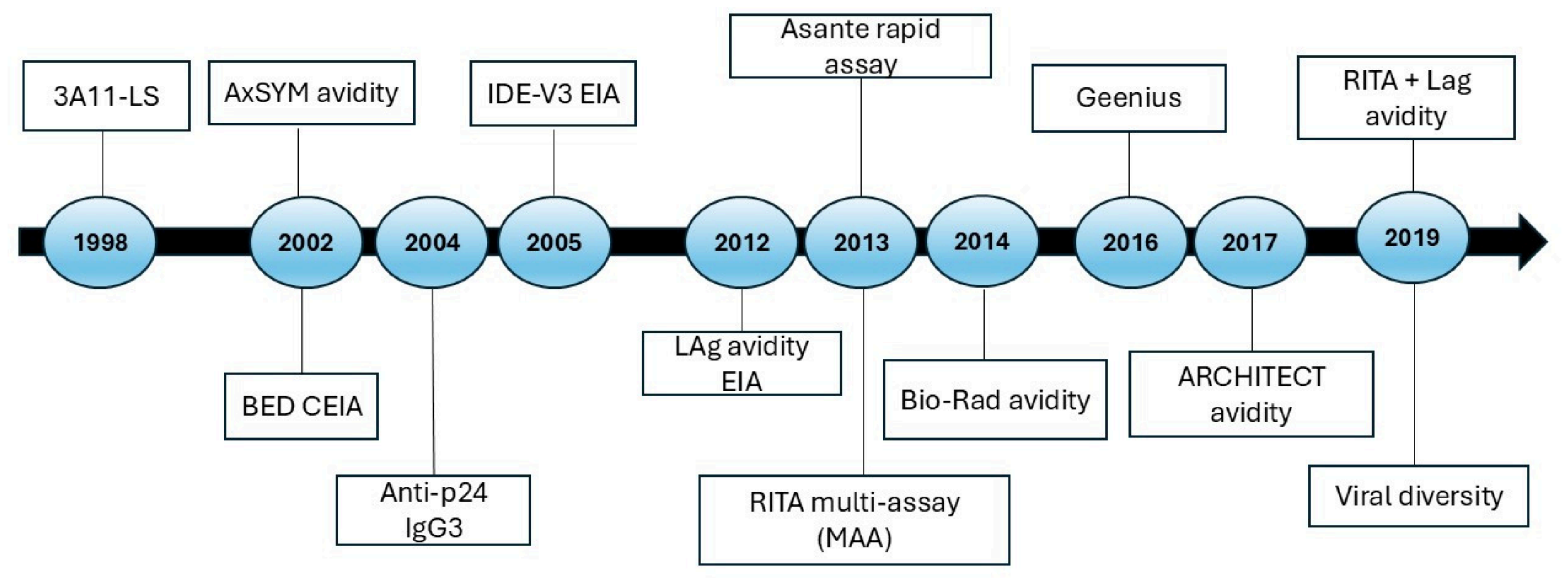
Timeline of HIV recency assays. The individual assays or their combination are described in the text, the boxes report each assay or methodology by the year of publication [5,6,7,9,10,12,23,24,25,26,27,28,29]. Abbreviations: Lag, limited antigen; EIA, enzyme immunoassay; CEIA, capture enzyme immunoassay; RITA, recent infection testing algorithm; MAA, multi-assay algorithm.

The limitations of testing for viral components (p24 antigen and/or HIV-RNA) have led research on recency testing to focus primarily on the host immune response to HIV infection, which matures over time [21,22]. The timeline for the development of the different diagnostic approaches is reported in Figure 4.

A detailed list of the most relevant diagnostic solutions (Table 2) includes the following:

- Western blot: This method, as well as other immunoblots, was originally conceived to confirm positivity for HIV-specific antibodies. Because positivity for the different antigen bands increases over time, it can help identify relatively recent HIV infections through profiling of specific antibody bands. However, its utility is limited by the relatively short window period during which early antibodies remain undetectable [19,22], resulting in limited sensitivity for HIV recency after 2–3 months from infection and frequent misclassification of recent infections [30,31].
- Detuned assay: This approach is based on reducing the sensitivity of standard ELISA/EIA antibody tests through sample dilution or altered incubation times. Recently infected individuals have lower HIV antibody levels and therefore may not react on a “tuned down” test. The MDRI is approximately 109–149 days [5]. The laboratory procedure is time-consuming and includes several manual steps that can lead to variability in results. It was primarily used for population-level epidemiological studies rather than individual diagnosis and was never commercialized.
- BED-CEIA (IgG-capture enzyme immunoassay): This assay measures the proportion of HIV-specific IgG, which is low during recent infection, relative to total IgG. It uses a synthetic branched peptide containing HIV-1 gp41 sequences from various HIV subtypes [6]. The MDRI varies between 160 and 180 days. It was the first commercially available recency test and was widely studied. However, it yields a high false-recent rate, depending on geographical and subtype variation, ART, and AIDS stage [32].
- Antibody avidity: This approach measures the overall strength of binding between antibodies and their specific target antigens [21], which is weak during early infection and stronger in established infections [7,8,9,10]. The MDRI can be modified by changing the cutoff value used to discriminate recent from established infections; the MDRI most frequently used corresponds to 6 months. According to the cited study, performance was not affected by elite controllers, samples with low viral load or CD4 counts, or HIV subtype [23]. False-recent results were observed in patients receiving early ART and in those with advanced disease. The methodology is relatively simple to perform and has been used in many laboratories in several countries, but only one commercial option is available [33].
- Subtype-specific IgG: An early, transient peak of IgG3 towards the p24 antigen of HIV-1 has been observed over an interval of 1–4 months after infection [24]. The presence of this reactivity might therefore help distinguish recent from established infections, but this approach was not further developed and is no longer used.
- IDE-V3 EIA: An ELISA was developed to quantify antibodies against four HIV-1 antigens: consensus peptides of the immunodominant epitope of gp41 (IDE), consensus V3 peptides, recombinant integrase, and recombinant p24. The IDE and V3 peptides were the best able to discriminate between the antibodies in serum from recently (<6 months) infected individuals and those with long-standing infection. Combined quantification of antibody binding to these two synthetic antigens allowed identification of recent infections [25]. Although promising, this approach was not further developed.
- Limiting antigen avidity enzyme immunoassay (LAg-Avidity EIA): This assay has been widely used to identify recent HIV infection. It measures the avidity of HIV-specific antibodies, with lower avidity indicating more recent infection. It uses a recombinant HIV antigen coated at limiting concentrations to detect low-avidity antibodies and has been standardized and validated for commercialization. The MDRI is 132–143 days [10] and varies significantly according to the prevalent viral clade [34]. However, it is susceptible to false-recent results in individuals receiving ART, in elite controllers, or those with advanced disease. It is commercialized in the United States and many other countries and is distributed for “Research Use Only”, sold directly to epidemiologists and research institutions. UNAIDS provides detailed guidance on incorporating LAg into recent-infection testing algorithms [18].
- Signal/cutoff ratio of a fourth-generation assay: This approach is based on the wide dynamic range of signal-to-cutoff values obtained with an HIV-1/HIV-2 antigen/antibody combination assay. The assay can serve a dual purpose by providing both HIV diagnostic test results and recency information, without additional testing or modifications to the assay protocol. The MDRI ranged from 103 to 445 days, increasing with higher cutoff values [35].
- Multiplex bead assays: Recent innovations include multiplex assays capable of simultaneously measuring multiple biomarkers, sometimes referred to as “fifth generation” HIV tests. For example, a triplex multi-bead assay detects HIV-1 p24 antigen, HIV-1 antibodies, and HIV-2 antibodies in a single well. These assays can distinguish recent from established infections with high sensitivity and specificity by measuring antibody binding and avidity against multiple antigens [12]. The MDRI is estimated to be between 159 and 240 days. They offer high throughput and multi-parameter measurement and may improve accuracy, but they require specialized equipment and laboratory capacity.
- Rapid recency assay (RTRI: rapid test for recent infection): These tests are third-generation assays (detecting antibodies but not antigens) that can distinguish early from long-term infection based on the strength and avidity of antibodies that mature over time after seroconversion. The MDRI is approximately 6 months [29,36]. They can also be used in community-based settings and at the point of diagnosis, representing a major advance in access to HIV testing. Zambia’s national program has implemented RTRI-based surveillance, demonstrating feasibility and public health value [37]. These tests can be readily integrated into routine testing, supporting real-time surveillance. Results are available within 20–40 min, but reactive samples require laboratory-based confirmation. Performance varies among individuals receiving ART, elite controllers, those with advanced disease, and high-risk groups [38].
- Geenius HIV 1/2: This is a single-use immunochromatographic assay that measures antibodies against multiple HIV-1/2 antigens and was developed for HIV-1/2 confirmation. However, quantitative reading provides information on the increasing concentration and diversity of antibody responses over time during seroconversion, and a quantitative threshold of recent HIV infection has been proposed to classify infections as “recent” or “non-recent”. The MDRI is approximately 179 days [26].
- Molecular and genomic approaches: These methods are based on various principles and, although not yet widely used in routine surveillance, include viral diversity analysis, genomic incidence approaches, CRISPR-based diagnostics, and next-generation sequencing. Their main value is that they characterize the accumulation and structure of HIV genetic diversity within the host that is fundamentally different from antibody maturation. HIV infection usually begins with a relatively limited number of transmitted/founder viruses. Because HIV reverse transcriptase is error-prone and viral replication is rapid, mutations accumulate, and distinct viral lineages emerge. Over time, immune selection, viral competition, recombination, and stochastic processes generate a progressively heterogeneous within-host viral population, or quasispecies. Consequently, recent infection tends to be associated with lower viral sequence diversity than established infection. Molecular/genomic approaches show promise for identifying recent infection [27], but sequencing costs, laboratory and bioinformatic requirements, assay standardization, and validation across diverse HIV subtypes and populations currently limit their use.

**Table 2:** Assay principle and recency window for ten different laboratory-based approaches to HIV recency testing. EIA, enzyme immunoassay; IgG, G class immunoglobulins; BED, HIV subtypes B, E, and D; LAg, limited antigen.

Assay/Approach	Assay Principle	MDRI *	FRR
**Detuned/less sensitive EIA**	Modified EIA with reduced antibody detection; early IgG is not detected	~134–199 days	3.6%, highly variable [5]
**BED Capture EIA**	Proportion of HIV-specific IgG to total IgG	~160–180 days	7% subtype B, 25% subtype non-B [9]
**AxSYM HIV 1/2 + avidity**	Third-generation immunoassay with IgG avidity protocol	~180 days	7.7% [8]
**LAg avidity EIA**	Limiting antigen + antibody avidity; Low signal = recent infection	~132–143 days	0.15–1.27% [10]
**Bio-Rad avidity assay**	Urea-based dissociation of antibodies to measure avidity	~120–160 days	2% subtype B, 6% subtype non-B [9]
**ARCHITECT HIV 1/2 Combo + HIV avidity**	Fourth-generation immunoassay with IgG avidity protocol	~120 days	1.52% [17]
**Asantè rapid recency assay**	Lateral flow assay with multisubtype recombinant gp41 protein at two concentrations (high and low)	~180 days	0.32% with strip reader [38]
**Geenius HIV 1/2 + recency interpretation**	Confirmatory immunoassay band patterns for recency	~179 days	4.1% (excluding treated individuals and elite controllers) [26]
**RITA multi-assay algorithms**	Combinations of serology, HIV-RNA viral load +/− CD4 count	~155–187 days	1.1–14.9% [17]

* MDRI is approximate and depends on population, HIV subtype, assay cutoff, and testing algorithm. The above data are reported from validation studies and CDC/WHO surveillance guidance.

## 6. Recent Infection Testing Algorithm (RITA)

**Figure 5:**
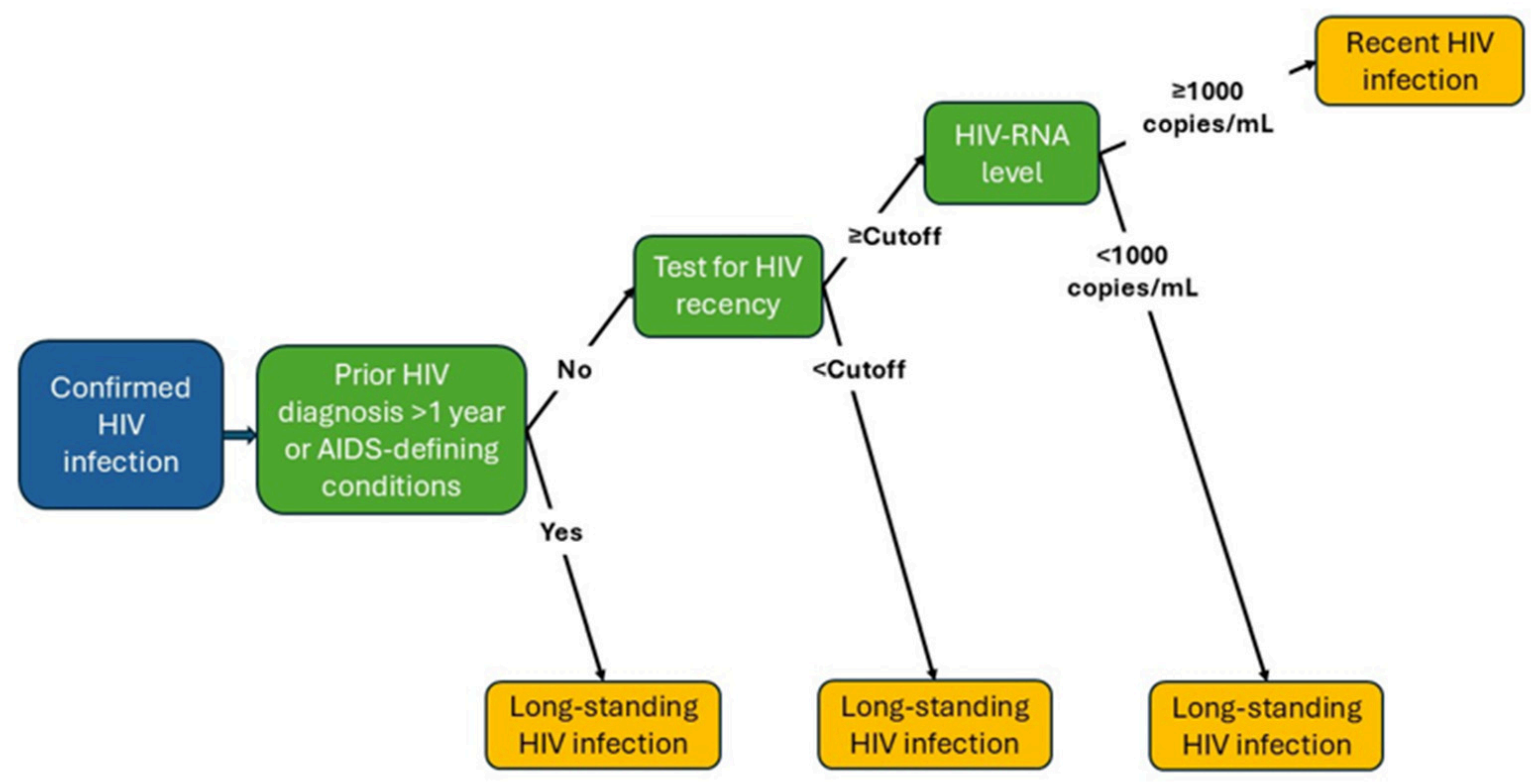
Example of the recent-infection testing algorithm (RITA) to distinguish recent from long-standing HIV infection.

Stand-alone assays frequently fail to achieve a sufficiently long MDRI while keeping the FRR below 2%, owing to biological variability and confounding factors. Consequently, modern approaches typically rely on RITAs (initially called the Serologic Testing Algorithm for Recent HIV Seroconversion—STARHS) [39], which combine avidity assays with additional laboratory and clinical data to filter out virally suppressed or long-term cases. The rationale of RITA is that additional markers can reduce the likelihood that a person with long-standing infection will be classified as recently infected. These algorithms are primarily used at the population level to estimate HIV incidence rather than for individual patient diagnosis. A RITA (Figure 5) combines the following:

- A laboratory assay (e.g., antibody avidity);
- Clinical information (e.g., ART status, clinical stage, AIDS diagnosis, PrEP);
- Viral load measurements;
- Additional biomarkers, when available (e.g., CD4 count).

For example, the combination of LAg avidity with a viral load threshold has been extensively investigated. Subsequent approaches have incorporated ARV drug detection to further reduce misclassification among individuals receiving antiretroviral therapy [16]. Depending on the parameters included, the MDRI ranges between 155 and 187 days [18]. A major advantage of RITAs is their ability to reduce the FRR by incorporating additional biomarkers and clinical criteria. In one study, the FRR decreased from 6.1% with BED alone to 5.6% with the Bio-Rad avidity assay, 2.3% with the combination of BED and the Bio-Rad avidity assay, and further to 1.43% when CD4 count and viral load criteria were added [40].

UNAIDS encourages the use of these algorithms to obtain more accurate incidence estimates by reducing false-recent results. Moreover, UNAIDS recommends contextualizing RITAs to local epidemiology, including the prevalence of early ART initiation, HIV subtype distribution, population mobility, and testing frequency, all of which can affect RITA results [18].

## 7. Applications for Recent Infection Testing

### 7.1. Population-Level Surveillance

**Figure 6:**
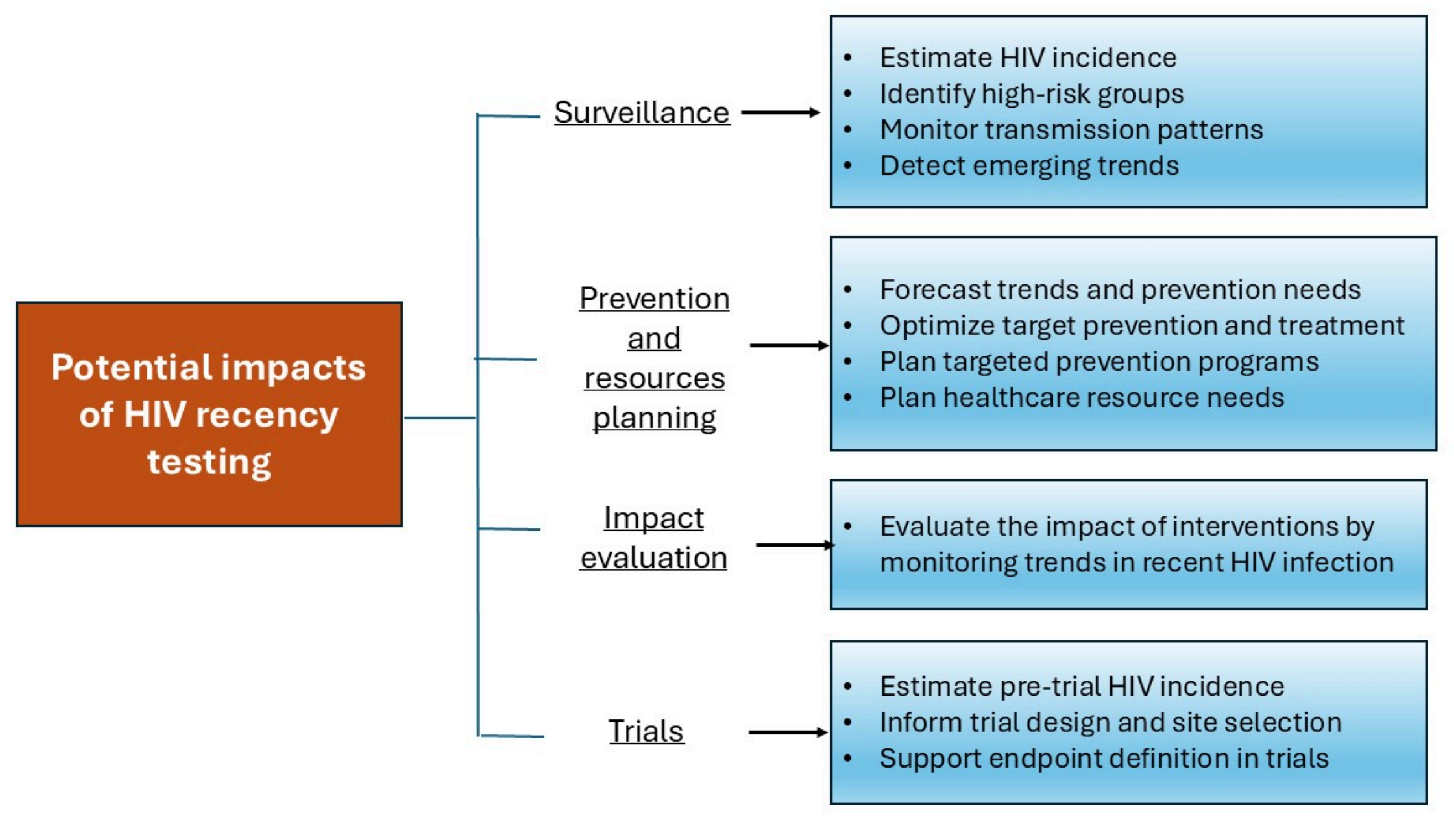
Conceptual framework of the potential public health and clinical impacts of HIV recency testing.

These methods are primarily used for public health surveillance rather than individual diagnosis [18,24,27,28,33,39,41]. They have potential value at several levels, including incidence estimation, identification of high-transmission hotspots, optimization of linkage to care, prevention interventions, and resource allocation [Figure 6].

For surveillance purposes, HIV incidence estimation is a key indicator for monitoring epidemic trends. Recent-infection assays can be used to estimate incidence based on new infections rather than new diagnoses (which are usually reported to national HIV surveillance systems). These incidence estimates are important for generating more accurate trend projections and transmission-risk models. At the population level, they are useful for population-based surveys and studies of key populations, providing cross-sectional incidence estimates without requiring longitudinal studies.

These tests can enable real-time identification of population groups or clusters within specific demographic or geographic areas with high transmission rates, thereby helping public health stakeholders plan targeted prevention strategies and enabling health workers to focus intervention resources on individuals most likely to be at the center of an active outbreak. Prevention strategies and resources can be more appropriately targeted Taksand allocated by concentrating efforts on key populations or areas with a high incidence of recent infection. Furthermore, recent infection testing can help evaluate the effectiveness of prevention programs, the impact of ART scale-up, and trends in undiagnosed infection.

### 7.2. Individual Level

While a prompt start of ART treatment is recommended regardless of the recency of infection, identifying a recent HIV infection may create an opportunity to engage patients in early ART initiation without being interpreted as a prerequisite for treatment initiation. Explaining the potential benefits of prompt treatment may support linkage to care and treatment adherence; early ART also limits the establishment and persistence of the viral reservoir and improves long-term clinical outcomes, specifically, better quality of life, fewer comorbidities, longer survival, and lower mortality. Moreover, information on the likely timeframe of infection can help healthcare providers offer more tailored counseling and education regarding viral suppression and treatment adherence. Contact-tracing activities can be prioritized to identify and test sexual or needle-sharing partners of newly infected individuals, with the aim of reducing onward transmission.

## 8. Strengths and Limitations of Recent Infection Testing

Recent infection testing has several strengths that make it potentially suitable for routine use, especially at the population level. Specifically:

- It enables cross-sectional incidence estimation without requiring burdensome and time-consuming longitudinal studies.
- It supports targeted public health interventions: this approach can help map sexual or drug-use networks to reach at-risk individuals who traditionally may not access HIV testing and can help optimize funding allocation for prevention strategies addressing high-risk populations.
- It integrates into routine testing workflows. The assay can be performed on the same specimen and platform used for the HIV screening test, with no additional specimen required for the assay procedure.
- It provides real-time surveillance data. Mapping the geographic and demographic distribution of recent infections can help health officials monitor the epidemic in real time and identify active outbreaks.
- It is increasingly standardized through UNAIDS guidance. Rather than standardizing the chemical design of individual tests, this guidance standardizes how, when, and where recency tests are implemented. The use of recency assays is recommended for cross-sectional population surveys, epidemiological mapping, and estimation of population-level incidence.

However, these assays have several limitations that should be considered:

- Need for algorithmic approaches rather than reliance on a single assay to reduce the FRR and optimize the MDRI.
- False-recent results due to ART, advanced HIV disease, or elite control: people living with HIV who are receiving treatment and have suppressed viral loads, who have AIDS, or who are elite controllers may produce antibody responses similar to those of newly infected individuals, leading to misclassification.
- Variability across HIV subtypes: certain subtypes may cause false-recency results by generating weaker or altered antibody maturation curves, occasionally leading to misclassification of established infections as recent.
- Laboratory capacity requirements: testing may rely on automated molecular platforms and high-throughput immunoassay analyzers. Standardization, quality assurance, and quality-control panels are required to support robust reagent supply chains and ensure accurate interpretation of both routine diagnostic and incidence-testing results.
- Limited timeliness of the public health response: if these tests are not implemented in routine surveillance, a rapid impact on ongoing transmission at the population level may be difficult to achieve.
- Point-of-care RTRIs may require additional time, on-site training in procedures such as finger-prick blood collection, and supportive supervision. Point-of-care testing may also be affected by lower or more variable accuracy, quality-control and quality-assurance challenges, connectivity issues, and differences in operator experience and training [42]. Quality-assurance and quality-control requirements should be comparable to those applied to laboratory-based methods [42,43].
- Ethical considerations regarding disclosure of recency status at the individual level: UNAIDS stresses caution in disclosing recency results to individuals because misclassification may cause distress or stigma and recommends against using recency testing as part of routine HIV testing services.
- Overall, some HIV recency technologies have fallen out of use because of limitations in standardization, robustness, or scalability. Avidity-based and limiting-antigen assays, together with selected rapid recency tests, have been more widely adopted because of their relative simplicity and compatibility with routine testing and surveillance programs. Important technical challenges remain, including differences across HIV subtypes, false-recent classifications among people with long-standing infection or those receiving ART, and interindividual variability in biomarker kinetics. Assay thresholds and recency algorithms must therefore be calibrated for the relevant epidemiological and population settings. Implementation in routine testing and surveillance systems is feasible but requires attention to specimen quality, laboratory workflows, staff training, result interpretation, and the integration of complementary biomarkers. In the near term, genomic approaches are more likely to complement than replace serological assays. By characterizing within-host viral diversity and quasispecies structure, genomic methods provide information partly independent of antibody maturation and may improve classification in challenging cases. However, their cost, complexity, and lack of standardization limit their use as stand-alone tools. The field is therefore moving toward scalable approaches that integrate serological, virological, and genomic biomarkers rather than relying on a single measure of recency.

## 9. Steps for Implementation

### 9.1. Population-Level Surveillance

Successful implementation of this strategy for surveillance purposes requires, first and foremost, agreement within the national laboratory network on the use of specific assays for recent infection and on the parameters and cutoffs to be incorporated into the algorithm so that results can be standardized and compared across services. Internal and external quality-assurance and quality-control procedures should be established in all laboratories to enable interlaboratory comparison.

Adequate training of laboratory staff should be planned to ensure competency in equipment operation and laboratory procedures and to prepare staff for the routine inclusion of reflex recency testing for all new HIV diagnoses.

The results of recency tests should be recorded in surveillance registers for epidemiological analysis, and appropriate data-transmission systems and infrastructure should be provided to enable real-time epidemiological information.

### 9.2. Individual Level

Future developments and improvements in these assays may lead to greater accuracy and precision in identifying recent infections at the individual level. In such scenarios, recency testing could be introduced into routine clinical practice to support individual clinical decisions, such as prioritizing partner notification or tailoring ART initiation pathways. If recency testing is introduced at the clinical level, the interpretation and communication of test results should be discussed and agreed upon by laboratory staff and healthcare professionals. Physicians should consider the use of test results in the context of individual epidemiological and clinical assessments, while ensuring respect for informed consent and patient autonomy. Decisions regarding the disclosure of results should take into account the patient’s right to be informed, their preferences, and the ethical and clinical implications of disclosure. When results are communicated, patients should be provided with tailored information and appropriate counseling, as well as support for contact tracing and partner notification, where appropriate.

Healthcare workers should receive tailored training in test interpretation and communication skills focused on the potential use of these results. Clear protocols for follow-up and intervention would help standardize healthcare approaches.

## 10. Ethical and Data Governance for HIV Recency Assays

Implementing HIV recency assays requires careful consideration to ensure that recency information is used responsibly and does not lead to misinterpretation, discrimination, or harm.

Consent: Individuals should be informed that recency testing may be performed on their HIV-positive specimen and that the result estimates the likelihood of recent infection rather than identifying the exact date of acquisition.

Disclosure: Results should be communicated carefully, with emphasis on their probabilistic nature. A result indicating recent or long-standing infection must not be interpreted as proof of when or from whom HIV was acquired.

Stigma: Recency testing may inadvertently reinforce HIV-related stigma or lead to blame, particularly if a result is interpreted as evidence that a specific individual recently transmitted HIV. Communication should avoid language implying responsibility or fault.

Confidentiality: Recency status is sensitive health information and should receive the same level of protection as other HIV-related information. Access to individual-level results should be restricted to authorized personnel.

Partner notification: Recency information may inform public health partner services and targeted prevention interventions, but its use should follow established confidentiality, consent, and safeguarding procedures.

Data governance: Because recency data may be linked to epidemiological, clinical, and molecular information, robust governance is essential. This includes clear rules for data ownership, access, linkage, storage, retention, secondary use, and reporting, as well as safeguards against re-identification.

Community engagement: Communities affected by HIV should be involved in designing and implementing recency-testing strategies. Such engagement can help ensure that terminology, consent procedures, communication, and data use are acceptable, understandable, and responsive to community concerns.

## 11. Conclusions

Tests for recent HIV infection have transformed the landscape of HIV surveillance and prevention. From laboratory-based avidity assays to rapid point-of-care tests, these tools provide critical insights into epidemic dynamics and help guide targeted interventions. While challenges remain, particularly regarding false-recent results and ethical considerations surrounding individual diagnosis, ongoing research and technological innovation continue to improve accuracy and feasibility.

UNAIDS guidance and recent scientific advances, including multiplex assays and national RTRI implementations, demonstrate a clear trajectory toward more precise, real-time, and actionable surveillance systems. As global HIV prevention efforts intensify, recent infection testing will remain a relevant component of public health strategy.

## Figures and Tables

**Table 1:** Rationale and limitations of laboratory biomarkers for recent HIV infections. ART, antiretroviral therapy; AIDS, acquired immune deficiency syndrome; PrEP, pre-exposure prophylaxis.

Biomarker	Rationale	Limitations
**Antibody avidity**	Antibodies bind progressively more strongly to antigens over time; low avidity suggests recent infection	Affected by ART, elite virus control, and advanced disease (late AIDS stage)
**Antibody concentration**	Total antibody levels increase during early infection	High variability between individuals
**Viral load**	Very high during acute infection	Not specific to recency; can be suppressed by ART or PrEP
**p24 antigen**	Detectable before antibodies	Short detection window; p24 becomes detectable also in late stages
**Molecular diversity**	Viral quasispecies increase and diversify over time	Complex and not suitable for routine surveillance

## Data Availability

No new data were created or analyzed in this study. Data sharing is not applicable to this article.

## Abbreviations

The following abbreviations are used in this manuscript:

HIV	Human Immunodeficiency Virus
WHO	World Health Organization
UNAIDS	The Joint United Nations Programme on HIV/AIDS
CEPHIA	Consortium for the Evaluation and Performance of HIV Incidence Assays
EIA	Enzyme Immunoassay
STARHS	Serological Testing Algorithm for Recent HIV Seroconversion
MDRI	Mean duration of recent infection
FRR	False-recent rate
ART	Antiretroviral therapy
EU/EEA	European Union & European Economic Area
ECDC	European Centre for Disease Prevention and Control
LAg	Limiting antigen
ARV	Antiretroviral
RITA	Recent infection testing algorithm
AIDS	Acquired immune deficiency syndrome
IDE	Immunodominant epitope
ELISA	Enzyme-Linked Immunosorbent Assay
RTRI	Rapid testing for recent infection
PrEP	Pre-exposure prophylaxis
CRISPR	Cluster Regularly Interspaced Short Palindromic Repeats
BED-CEIA	BED capture enzyme immunoassay
